# Spatial genomics uncovers cytokines promoting ovarian tumour heterogeneity and immunotherapy resistance

**DOI:** 10.1002/ctm2.70248

**Published:** 2025-02-23

**Authors:** Gurkan Mollaoglu, Brian D. Brown, Alessia Baccarini

**Affiliations:** ^1^ O'Neal Comprehensive Cancer Center, Immunology Institute University of Alabama at Birmingham Birmingham Alabama USA; ^2^ Department of Microbiology, Heersink School of Medicine University of Alabama at Birmingham Birmingham Alabama USA; ^3^ Icahn Genomics Institute, Precision Immunology Institute Tisch Cancer Institute New York New York USA; ^4^ Department of Immunology and Immunotherapy Icahn School of Medicine at Mount Sinai New York New York USA

**Keywords:** immunotherapy, IL‐4, ovarian cancer, tumour immunology

1

Ovarian cancer (OvCa) is a leading cause of cancer‐related deaths among women, with a five‐year survival rate of less than 50%.^1^ Despite a woman's lifetime risk of developing OvCa being as high as one in 91, the lack of effective screening methods and the disease's subtle, nonspecific symptoms—often mistaken for benign conditions—result in most cases being diagnosed at advanced stages. Standard treatment for advanced OvCa includes a combination of debulking surgery and chemotherapy, with some patients also receiving targeted therapies such as Bevacizumab (a vascular endothelial growth factor inhibitor) or Olaparib (a poly[ADP‐ribose] polymerase inhibitor). However, even with optimal surgery and chemotherapy, most tumours recur within 18–24 months, often developing resistance to further treatment.^1^ To date, immunotherapies have shown limited success in OvCa, with clinical trials using immune checkpoint inhibitors reporting objective response rates below 10%.^2^ This is despite the moderate tumour mutation burden and PD‐L1 positivity observed in OvCa. Increasing evidence from preclinical and clinical studies suggests that OvCa's highly immunosuppressive tumour microenvironment is responsible for the failure of immunotherapy.^3^

Ovarian cancer is a prime example of intratumoral heterogeneity (ITH), a key driver of treatment failure across many cancers.^4^ Ovarian tumours almost universally exhibit *TP53* loss, along with frequent somatic and germline mutations in homologous recombination repair pathway genes—most notably *BRCA1* and *BRCA2*—leading to homologous recombination deficiency in approximately half of the cases. Without proper DNA repair mechanisms, these tumours accumulate extensive chromosomal abnormalities, including copy number variations and structural alterations, resulting in profound genomic instability.^5^ The long latency of tumour development, coupled with widespread metastatic dissemination to peritoneal organs, provides fertile ground for OvCa to grow with significant ITH, which in turn promotes immune evasion and treatment resistance.

Understanding how ITH drives immune evasion and immunotherapy resistance is therefore of paramount importance. Notably, ITH is not confined to cancer cells but also manifests within the tumour microenvironment (TME), influencing immune cell abundances, functional states, and cellular interactions.^6^ A fundamental question is how tumour clones establish their distinct TMEs and to what extent clonal TME influences clonal selection. Sequencing and imaging‐based studies of clinical samples have identified certain genomic correlates of ovarian TME phenotypes, such as genetic alterations affecting angiogenesis, antigen presentation, oxidative phosphorylation, and inflammatory signalling pathways.^7^, ^8^, ^9^, ^10^ However, establishing a causal link between dysregulated cancer genes and immunity, especially in the context of tumoral heterogeneity, remains challenging.^11^ To directly test the functional roles of genes and pathways implicated by human OvCa TME studies, mouse models that can recapitulate the ITH observed in patient tumours are required. Furthermore, studying clonal TMEs requires spatial resolution of heterogeneous tumours to link each clone's genotype with its corresponding microenvironment.

In a recent study published in *Cell*,^12^ we employed Perturb‐map, a spatial functional genomics tool we previously developed,^13^ to model and spatially resolve ITH in an animal model of metastatic OvCa. Perturb‐map is a novel technology that uses protein barcodes^14^ to enable CRISPR screens to be read within a tissue by imaging, as well as spatial transcriptomics,^13^ bringing functional genomics into the spatial era. Using Perturb‐map, we investigated whether cancer cell‐extrinsic factors—such as receptor and ligand signalling molecules expressed on the cancer cell surface or secreted—can drive clonal immune selection by shaping distinct TMEs. We prioritized and functionally tested 34 genes implicated in cancer cell–TME communication.

Our Perturb‐map analysis revealed that cancer cells with each targeted CRISPR knock‐out (KO) grew clonally, leading to extensive ITH across metastatic sites (Figure 1). Interestingly, the loss of the *Plaur* gene completely abrogated the growth of OvCa clones in vivo, despite the fact that *Plaur* KO had no effect on the cancer cells in vitro. *Plaur* encodes for urokinase‐type plasminogen activator receptor (uPAR).^15^ As *Plaur* is highly expressed in many solid tumours, it has been explored as a therapeutic target, including chimeric antigen receptor (CAR)‐T cell target, across several cancers, including OvCa.^16^ Our data indicates that OvCa cells cannot grow in vivo without plasminogen activator urokinase receptor, and this provides an even stronger rationale for targeting it, as therapeutic escape may be less likely.

**FIGURE 1:**
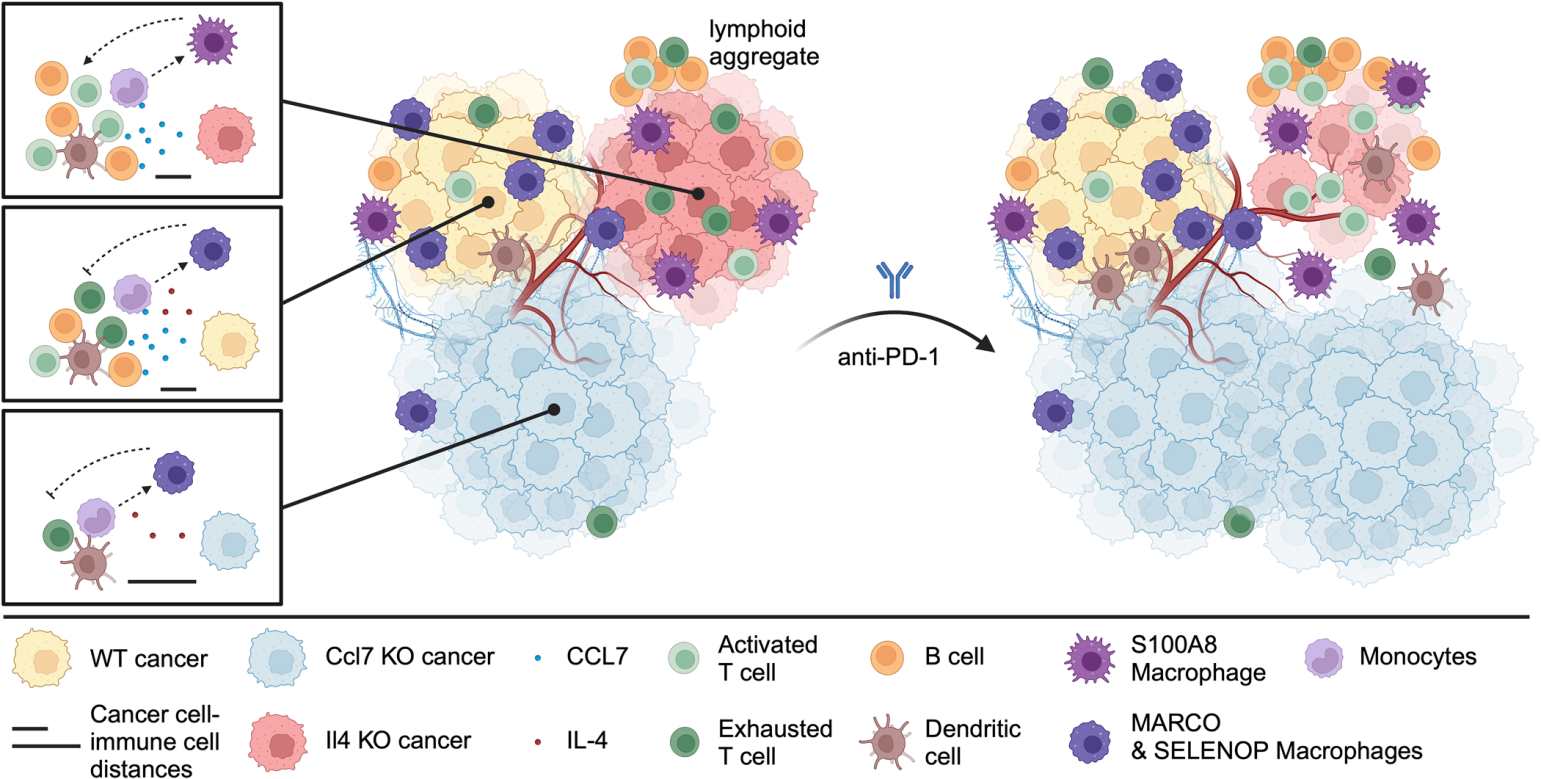
Spatial functional genomics of ovarian tumours revealed that cancer cell‐secreted cytokines shape clonal tumour microenvironments (TMEs), driving clonal immune selection. Tumour clones lacking chemokine (C‐C motif) ligand 7 (CCL7) develop immunologically cold TMEs, become more resistant to immunotherapy, and exhibit aggressive growth. Additionally, ovarian cancer cell‐secreted interleukin (IL)‐4 promotes macrophage receptor with collagenous structure (MARCO) macrophages, fostering an immunosuppressive TME that enhances immunotherapy resistance. Blocking cancer cell‐macrophage IL‐4 signalling, either genetically or pharmacologically, reshapes the TME by shifting macrophages toward an S100A8 phenotype, increasing lymphoid aggregate formation, and enhancing T cell infiltration—ultimately rendering these clones sensitive to immunotherapy.

Perturb‐map also found that KO of *Ccl7* created tumour clones with a very aggressive growth advantage. We subsequently demonstrated that the loss of chemokine (C‐C motif) ligand 7 (CCL7) cytokine created immunologically “cold” tumours with significantly reduced immune cell infiltration, impairing the recruitment of multiple immune cell types. CCL7 is a secreted cytokine, and while *Ccl7* KO clones coexisted with *Ccl7* wild‐type clones within the same tumour masses, each exhibited distinct TMEs (i.e. immune‐desert vs. immune‐infiltrated, respectively), granting a significant clonal selection advantage to the former (Figure 1). Consistently, we found that CCL7 protein was spatially restricted to *Ccl7* WT clones, demonstrating that a cancer cell‐secreted cytokine can locally regulate clonal TME within heterogeneous tumours. In OvCa patients, *CCL7* expression appears to be progressively lost with the cancer stage, and its levels can even serve as a predictor of patient survival. These findings suggest that tumour clones with lower *CCL7* expression and reduced immune infiltration gain a selective advantage by evading immune surveillance.

Next, we investigated whether any of the selected cancer cell–TME signalling molecules influenced immunotherapy response. Using Perturb‐map, we compared anti‐programmed cell death protein 1 (anti‐PD‐1)‐treated and control tumours in the ID8 model and identified *Ccl7* and *Il4* genes as key regulators of immunotherapy response. Loss of *Ccl7* conferred resistance to immunotherapy, consistent with its immune‐desert TME. Conversely, *Il4* KO clones were significantly diminished upon treatment, indicating that cancer cell‐secreted interleukin (IL)‐4 promotes resistance to anti‐PD‐1 therapy. Spatial proteomics and single‐cell transcriptomics revealed that the cancer cell uses IL‐4 to program a specific subset of macrophages, called macrophage receptor with collagenous structure (MARCO) macrophages, and these protect the cancer cells from T cells, even when animals are treated with anti‐PD‐1 immunotherapy (Figure 1).

These findings highlight key aspects of IL‐4 biology. First, cancer cells can produce IL‐4, and while this may be in small amounts and not the only source of IL‐4 in the tumour, cancer cell‐secreted IL‐4 exerts a potent influence on the TME. Second, cancer cell‐secreted IL‐4 specifically reprograms macrophages to promote the MARCO phenotype, which further shapes the TME, amplifying the effect of cancer cell‐secreted IL‐4. Third, while blocking IL‐4 signalling between cancer cells and macrophages significantly alters the TME, its role in clonal selection becomes crucial only in the context of immunotherapy treatment.

Finally, we tested the therapeutic potential of targeting IL‐4 signalling in a preclinical setting. We found that a combination of anti‐PD‐1 plus anti‐IL‐4 receptor (IL4R) therapy produced a potent anti‐tumour effect and significantly improved survival, whereas either treatment alone had no efficacy. This is an exciting finding as there is already an FDA‐approved drug which blocks IL4R, Dupilumab, that is used to treat atopic diseases like asthma. Our results support the rationale for testing anti‐IL4R in combination with anti‐PD‐1 inhibitors (e.g. Pembrolizumab or Nivolumab) in clinical trials for OvCa treatment. Since Dupilumab is already in clinical use for conditions such as eczema and asthma, translating this combination immunotherapy to the clinic could be expedited.

Our study demonstrates that cancer cells produce IL‐4 to create a spatially protected environment for themselves, driving clonal resistance to immune checkpoint blockade (ICB). Recent findings indicate that IL‐4 mediates ICB resistance in lung cancer and contributes to CD8 CAR‐T cell therapy resistance in lymphoma.^17^, ^18^ Conversely, another study suggests that IL‐4 can enhance adoptive T cell transfer or ICB efficacy in melanoma and colon cancer models.^19^ These findings highlight the context‐dependent role of IL‐4 in tumour immunity, potentially reflecting variations in TME across different cancer types. While further research is needed to fully elucidate IL‐4′s role in tumours, targeting IL‐4 signalling is emerging as a promising new strategy in cancer immunotherapy.

## AUTHOR CONTRIBUTIONS

Gurkan Mollaoglu, Brian D. Brown and Alessia Baccarini contributed equally to the writing and editing of the letter.

## CONFLICT OF INTEREST STATEMENT

Brian D. Brown has a patent application on the Pro‐Codes, which have been licensed to Immunai and Noetik.

## FUNDING INFORMATION

B.D.B. is supported by R01CA254104 and funding from the Feldman Foundation.

## ETHICS STATEMENT

Not applicable.
